# Estimated and forecasted trends in domain specific time-use and energy expenditure among adults in Russia

**DOI:** 10.1186/1479-5868-11-11

**Published:** 2014-01-30

**Authors:** Tracy Dearth-Wesley, Barry M Popkin, Shu Wen Ng

**Affiliations:** 1 Department of Nutrition and Carolina Population Center, University of North Carolina at Chapel Hill, Chapel Hill, NC, USA

**Keywords:** Physical activity, Time-use, Sedentary, Active transport, Movement, Russia

## Abstract

**Background:**

Examination of historical trends and projections in estimated energy expenditure in Russia is important given the country’s economic downturns and growth.

**Methods:**

Nationally representative data from the Russia Longitudinal Monitoring Survey (RLMS) from 1995–2011 was used to determine the metabolic equivalents of task (MET)-hours per week from occupational, domestic, travel, and active leisure physical activity (PA) domains, as well as sedentary leisure time (hours per week) among adults 18–60 years. Additionally, we projected what these values would be like in 2020 and 2030 if observed trends continue.

**Results:**

Among male adults, the largest contributor to total PA was occupational PA followed by travel PA. In contrast, domestic PA followed by occupational PA contributed most to total PA among female adults. Total PA was 282.9 MET-hours per week in 1995 and declined to 231.7 in 2011. Total PA is projected to decrease to 216.5 MET-hours per week in 2020 and to 193.0 MET-hours per week in 2030. The greatest relative declines are occurring in travel PA. Female adults are also exhibiting significant declines in domestic PA. Changes in occupational and active leisure PA are less distinct.

**Conclusions:**

Policies and initiatives are needed to counteract the long-term decline of overall physical activity linked with a modernizing lifestyle and economy among Russian adults.

## Background

Initiatives designed to reduce the global burden of overweight and obesity require understanding of environmental and individual factors affecting dietary and physical activity (PA) patterns and monitoring of these patterns over time and across countries [1-4]. With respect to PA, the International Physical Activity Questionnaire (IPAQ) and the Global Physical Activity Questionnaire (GPAQ) enable surveillance of PA and international comparisons [5-7]. More rigorous examination of PA, such as more detailed time allocation and energy expenditure in domain-specific activities, can be achieved through utilization of longitudinal and cross-sectional country-specific datasets [8-12]. Past analyses of country-specific data from the United States, the United Kingdom, China, Brazil and India have described historical trends in estimated average energy expenditure in four domains of activity (occupation, domestic production, travel and active leisure) and sedentary time in adults, and also projected changes in energy expenditure in these domains and sedentary time for 2020 and 2030 [9]. Extension of this research on historical trends and projections in energy expenditure to include Russia, a country that ranks 9th in the world by population (~143 million people) [13], would strengthen the research base for more thorough international PA comparisons and contribute to more effective domain-specific initiatives [9].

Examination of historical trends and projections in estimated energy expenditure in Russia is additionally important given the country’s economic downturns and growth. The Russian economy suffered a major depression in the early to mid-1990s, with a brief recovery in 1996–7, only to face a serious financial crisis in 1998. Following this crisis, the economy recovered for the next 10 years, posting gross domestic product growth ranging from 4.7 to 10.0% [14,15]. After a smaller recession in 2008–2009, the economy is recovering [16]. While some research has looked at the impact of these economic transitions on dietary patterns [17-19], less is known about how these transitions influenced PA across the domains and what can be expected in the next 10–20 years. PA projections not only provide valuable insight into potential PA patterns if no actions are taken but also help prioritize the development and implementation of domain-specific PA initiatives.

Particular focus on understanding how these economic transitions influence occupational PA is key, given occupational PA is a primary contributor to total PA [9]. Additionally, the Russian dataset includes occupational data that measures both the time and intensity of occupational activities (e.g., time spent in a usual workday doing moderate physical effort while standing or in movement), thus providing a unique opportunity to compare three distinct approaches for determining metabolic equivalents of task (MET) values for occupational PA. While the first approach involves assigning MET values to occupations or occupational categories using the Compendium of Physical Activities [20], the second and third approaches utilize different measures of time and intensity from occupational activities to determine MET values for occupational categories. Comparison of these approaches would yield methodological evidence important for determining a robust approach for measuring occupational PA.

There have been limited analyses of child PA patterns in Russia, and little research has been conducted on adults [21,22]. Using cross-sectional data from the nationally representative Russia Longitudinal Monitoring Survey (RLMS), we examined PA patterns in male and female adults (18–60 years) over a 16-year time period (1995 to 2011). PA patterns included 4 activity domains (occupation, domestic production, travel, and active leisure) and sedentary time. Our primary study objectives were to (1) compare three approaches for determining MET values for occupational PA, (2) estimate average energy expenditure for the activity domains and sedentary time and look at changes over time, and (3) forecast estimated average energy expenditure for PA domains in 2020 and 2030.

## Methods

### Data

The RLMS is a de-identified publicly available data source that includes a series of nationally representative, household-based surveys developed to examine the effects of Russian reforms on the health and economic well-being of households and individuals in the Russian Federation [23-26]. A multi-stage probability sample was used. While the RLMS was not specifically designed to examine PA, participants were asked to report on the frequency and duration of various activities across occupation, domestic production, travel, active leisure, and sedentary domains. Some Rounds of the RLMS also asked about the intensity of occupational activities. Data from RLMS Rounds 6 to 20 were analyzed, spanning a 16-year time period including surveys conducted in 1995, 1996, 1998, and 2000–2011. The number of sampled households was approximately 4,000 for Rounds 6 to 18 (1995–2008) and increased to approximately 6,000 for Rounds 19 and 20 (2010–2011).

### Estimating average energy expenditure for PA domains

Estimated averages of energy expenditure among adults in Russia were determined for 4 PA domains: occupational, domestic, travel, and active leisure. Additionally, we attempted to estimate sedentary leisure time per week for a subset of the adult population based on available data (Rounds 10–11). This subset included adults who previously participated in the RLMS Child Survey, in which time spent watching television or videos was reported. Note that we do not account for time spent during and energy expended from sleep or personal/self-care activities, as time spent sleeping was only measured in Rounds 5–8 and personal/self-care activities were not measured in the RLMS surveys.

Occupational PA included self-reported measures of time spent in primary and secondary occupations. Determination of estimated MET values for these occupations was done using three approaches. For all three approaches, the occupations were first coded into 10 main categories (e.g., professionals, clerks, service and market workers, etc.), according to the International Standard Classification of Occupations: ISCO-88 [27]. The ISCO classification of jobs in the RLMS was previously determined using computer and coder analyses of responses to various occupation questions along with careful consideration of the Russian labor market [28]. Following the categorization of occupations, the most frequently reported occupations within each occupational category were determined (Table 1). Using this information, the first approach (Approach A) assigned MET values to these occupations or more generally to the occupational category using the Compendium of Physical Activity [20]. The MET values within each occupational category were then averaged to determine a MET value for each occupational group (Table 1). This approach for MET value assignment was necessary given the previously determined ISCO classifications; comparison with other approaches, such as that developed for the American Time Use Survey [29], was done where there was some general overlap in the main occupational categories.

**Table 1:** **Occupational categories, frequently reported occupations, compendium codes and descriptions, and average MET values based on 3 approaches**^**1**^

**Occupational categories**	**Most frequently reported occupations**	**2011 Compendium codes and descriptions**	**2011 MET value**	**Average MET value**		
				**Approach A**^**2**^	**Approach B**^**3**^	**Approach C**^**4**^
Legislators, Senior Managers, Officials	General Mgr, not classified (28.7%)	11472 manager, property	1.8	2.3	2.5	2.5
	General Mgr Wholesale (18.5%)	11585 sitting meetings, light effort, general	1.5			
	Other Dept Mgr (13.2%)	11792 walking on job, 3.0 mph, moderate speed	3.5			
Professionals	Architect/Engineer, not classified (13.0%)	11135 engineer (e.g., mechanical/electrical)	1.8	2.3	2.1	2.4
	Teachers (13.9%)	11585 sitting meetings, light effort, general	1.5			
	Doctors (9.0%)	11792 walking on job, 3.0 mph, moderate	3.5			
Technicians and Associate Professionals	Bookkeepers (18.0%)	11610 standing, light/moderate effort (e.g., nursing)	3.0	2.7	2.7	2.5
	Nurses (13.5%)	11580 sitting tasks, light effort	1.5			
	Technicians, not classified (5.6%)	11792 walking on job, 3.0 mph, moderate	3.5			
Clerks	Store Clerks (22.9%)	11600 standing tasks, light effort (e.g., store clerk)	3.0	2.3	2.3	2.2
	Cashiers (12.5%)	11580 sitting tasks, light effort (e.g., office work)	1.5			
	Secretary (11.7%)					
Service and Market Workers	Shop salespersons (47.2%)	11600 standing tasks, light effort (e.g., store clerk)	3.0	4.0*	3.8	3.7
	Police officers (10.3%)	11528 police, making an arrest, standing	4.0			
	Cooks (10.3%)	11115 cook, chef	2.5			
	Stall/market salespersons (8.0%)	11060 carrying moderate loads upstairs, moving boxes	8.0			
Skilled Agricultural and Fishery Workers	Forestry workers and loggers (35.0%)	11264 forestry, moderate effort	4.5	4.7	4.8	3.5
	Market-oriented crop/animal producer (12.7%)	11192 farming, taking care of animals, general	4.5			
	Market-oriented animal producer, not elsewhere classified (10.2%)	11146 farming, moderate effort	4.8			
		11248 fishing, commercial, moderate effort	5.0			
Craft and Related Trades	Agricultural/industrial-machinery mechanics (15.9%)	11450 machine tooling, moderate effort	5.0	3.8	5.0	3.7
	Welders (10.3%)	11430 machine tooling (e.g., welding)	3.0			
	Mechanics (8.5%)	11420 locksmith	3.0			
	Locksmith (7.4%)	11040 carpentry, general, moderate effort	4.3			
	Carpenters (6.7%)					
Plant and Machine Operators and Assemblers	Heavy truck and lorry drivers (16.5%)	11766 truck driving, loading and unloading	6.5	4.0	4.0	3.1
	Driver (12.1%)	11610 standing, moderate effort (e.g., assemble heavy parts)	3.0			
	Motorized farm/forestry operator (10.8%)	11500 operating heavy duty equipment, automated	2.5			
Elementary (Unskilled) Occupations	Domestic helpers/cleaners (23.0%)	11126 custodial work, moderate effort	3.8	4.4	4.7	3.6
	Building caretakers (20.4%)	11476 manual/unskilled labor, general moderate effort	4.5			
	Farmhand/laborers (17.5%)	11146 farming, moderate effort	4.8			
Army	Armed forces	11585 sitting meetings, light effort, general	1.5	2.5	2.6	2.9
		11792 walking on job, 3.0 mph, moderate	3.5			

^1^Russia Longitudinal Monitoring Survey.

^2^Used MET values for occupations included in the 2011 Compendium of Physical Activities.

^3^Used significant and medium physical effort and sitting measures from a usual workday.

^4^Used sitting, standing, and walking measures from a usual workday.

*An average value for standing tasks and carrying moderate loads upstairs was determined. This value (5.5) was averaged with MET values for police and cook to determine the average MET value for service and market workers.

The second approach (Approach B) for estimating MET values for occupational categories utilized time and intensity measures for work activities from RLMS Rounds 6 to 11 (1996 to 2002). In these surveys, participants were asked about time spent in a usual workday from heavy and medium physical effort (while standing or in movement) and from sitting. The reported time spent in each work activity was multiplied by the associated MET value (e.g., 6.5 MET value for heavy physical effort based on the Compendium code: 11830 walking or walk downstairs or standing, carrying objects about 50 to 74 pounds). The total MET-hours per day was calculated by summing the MET values from heavy and medium physical effort and sitting, and this value was divided by the total hours working per day to get an estimated MET value per hour for the occupation. Average MET values per hour were determined for each occupational category and are shown in Table 1.

The third approach (Approach C) also used time and intensity measures for work activities from the RLMS Rounds 6 to 11 (1996–2002). These surveys asked participants about time spent in a usual workday from sitting, standing, or walking (not carrying a load). The reported time spent in each work activity was multiplied by the associated MET value (e.g., 1.5 MET value for sitting based on the Compendium code: 11585 sitting meetings, light effort, general). The total MET-hours per day was calculated by summing the MET values from sitting, standing, and walking, and this value was divided by the total hours working per day to get an estimated MET value per hour for the occupation. As with the previous two approaches, average MET values per hour using Approach C were determined for each occupational category and are included in Table 1.

Comparison of the three approaches showed consistency across almost all occupational categories. In comparing Approaches A and B, MET values for all but one occupational category were within <0.3 METs of each other (Table 1). For the “Craft and related trades” occupational category, the average MET value using Approach A was lower than Approach B (3.8 and 5.0, respectively). More variation was seen in the average MET values as determined by Approach C versus those from Approaches A and B. In particular, lower average MET values were found from Approach C for the more labor-intensive occupational categories (e.g., skilled agricultural and fishery workers, plant and machine operators and assemblers). These lower average MET values were expected, given the walking variable used in Approach C measures walking not carrying a load. Therefore, carrying heavier loads typical of these more labor-intensive occupations is not accounted for in Approach C.

To determine estimated energy expenditure for occupational PA, the average MET values from Approach B were used. This approach was chosen given its incorporation of time and more complete intensity measures of occupational PA specific to our sample population. The average MET-hours for each occupational category were multiplied by weekly measures of time spent in primary and secondary occupations. For primary occupations, total MET-hours per day were multiplied by 5 to derive the MET-hours per week measure. The 5-day work week measure for primary occupation was determined using RLMS data and examining the ratio of reported hours in a usual work week to reported hours in a usual work day (i.e., average ratio across survey years was 4.97). For secondary occupations, the total hours per week measure was determined by dividing the reported secondary work hours in the last 30 days by four; this value was multiplied by the MET-hours per day value from secondary work.

For the domestic, travel, and active leisure domains, self-reported measures of time spent in the different domains were multiplied by appropriate estimated MET values using the Compendium of Physical Activity [20]. Due to limitations on the questions asked, travel PA only included walking and did not include other modes of travel such as bicycling, taking public transit or driving. Domestic and active leisure PA included various subdomain activities. Subdomain activities for domestic PA consisted of preparing food, washing dishes, cleaning, looking for/purchasing food, laundry, child care, helping parents or relatives, and working on land or garden plot. Subdomain activities for active leisure PA included ball sports, jogging, swimming, ice-skating, skiing, exercise equipment, dancing, aerobics, karate, and boxing. The formula for determining the domain-specific MET-hours per week is as follows:

$${\left(\mathrm{Domain}\;\mathrm{MET}‒\mathrm{hours}\;\mathrm{per}\;\mathrm{week}\right)}_{a,i}\;=\;\sum_{s=1}^{s}\;{\mathrm{Time}}_{s,i}\;\times \;{\mathrm{MET}}_{s,i},$$

where *i* denotes an individual, *a* denotes PA domains, and *s* denotes subdomains. As for sedentary leisure time, the RLMS only asks about time spent watching television or videos and so we were unable to account for other sedentary leisure activities such as reading, listening to music, etc. (all while sitting).

Following the determination of the MET-hours per week from individuals for occupational, domestic, travel, and active leisure domains, as well as sedentary leisure time, weighted averages were determined for each RLMS Round. Post-stratification weights for individuals that fit the data to the multivariate distribution of location, age, and gender were used. Average values by Round were determined for adults 18-60 y and stratified by gender.

While data for occupational PA was available across all RLMS Rounds, data for domestic, travel, active leisure and sedentary leisure was less complete. Data on domestic PA was available for Rounds 6–8 (1995, 1996, 1998) and Rounds 15–18 (2006–2009); travel PA data was only available for Rounds 6–14 (1995–2005); active leisure PA data was available for all RLMS Rounds except Round 16 and 17 (2007–2008). Meanwhile, data on sedentary time was only available in two Rounds (Rounds 10 and 11) from 2001 and 2002. Therefore, with the exception of sedentary time, linear interpolation was conducted to determine average values for the missing Rounds across the activity domains.

### Changes in PA over time

Measures of change were calculated for all PA domains. The annualized change between time 1 (1995) and time 2 (varies by PA domain) was calculated by dividing the difference in the MET-hours per week between the two time points by the number of years between the two time points. The total percent change between time 1 and time 2 was determined by dividing the change between time 1 and time 2 by the time 1 MET-hours per week value; the result was multiplied by 100 to get a percentage. Lastly, the annualized percent change between time 1 and time 2 was determined by dividing the total percent change by the number of years between the two time points.

### Forecasting into 2020 and 2030

Estimated levels of PA for each domain in 2020 and 2030 were determined using three approaches: (a) using the slope from the last six Rounds of data (2006–2011) only; (b) using the slope from the last four Rounds of data (2008–2011) only, which included 2 years of economic downturn followed by two years of economic growth; (c) using three-year moving averages. The approach using the slope from the last six or four Rounds of data is based on the assumption that trends over time are linear. The total percent change between 1995 and 2020 or 2030 was also determined by dividing the change from 1995 to 2020 or 2030 by the 1995 MET-hours per week value; the result was multiplied by 100 to get a percentage.

## Results

**Figure 1:**
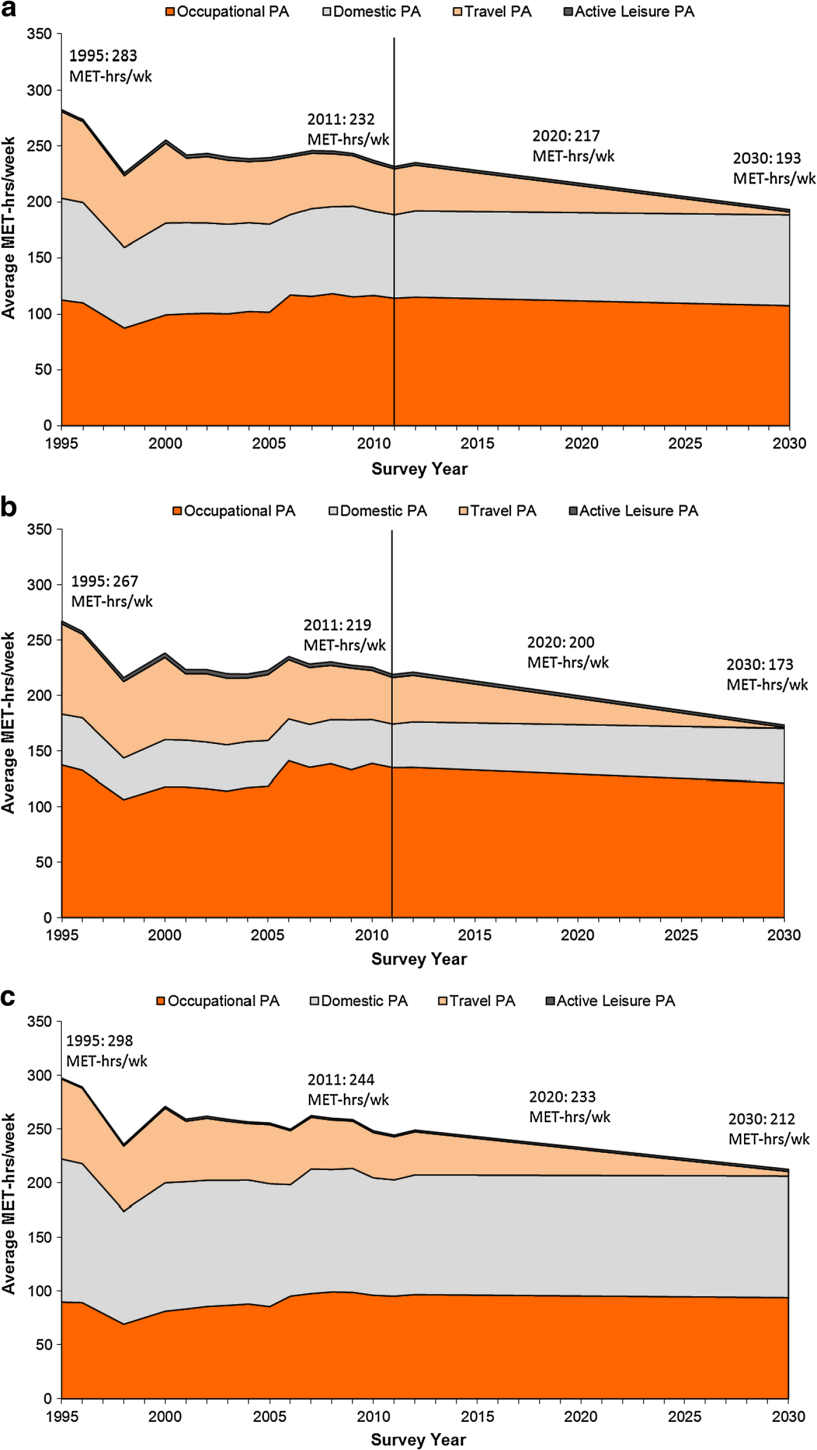
**Average MET-hours per week from PA for adults (18-60 y) and by gender for RLMS 1995-2011 and forecasted for 2020-2030. a**. Average MET-hours/week from PA for Adults (18–60 y) for RLMS 1995–2011 and forecasted for 2020–2030. **b**. Average MET-hours/week from PA for Males (18–60 y) for RLMS 1995–2011 and forecasted for 2020–2030. **c**. Average MET-hours/week from PA for Females (18–60 y) for RLMS 1995–2011 and forecasted for 2020–2030.

Average MET-hours per week for all PA domains from 1995 to 2011 and forecasted for 2020 and 2030 for Adults (18-60 y) are shown in Table 2. The same estimates are shown graphically in Figure 1a-1c. Total PA was 282.9 MET-hours per week in 1995 and declined to 231.7 in 2011. Total PA is projected to decrease to 216.5 MET-hours per week in 2020 and to 193 MET-hours per week in 2030. Among male adults, occupational PA followed by travel PA constituted the greatest components of total PA from 1995 to 2011. In contrast, domestic PA followed by occupational PA contributed most to total PA among female adults from 1995 to 2011. MET-hours per week from active leisure were relatively low for both genders. Average weekly time spent in each domain by gender is included in Table 3.

**Table 2:** **Average MET-hours per week for activity domains from RLMS 1995 to 2011 and forecasted for 2020 and 2030**^**a **^**for adults (18-60 y) by gender**^**b**^

**Activity domain**			**Average MET-hours per week by survey year**																
	**1995**	**1996**	**1997**	**1998**	**1999**	**2000**	**2001**	**2002**	**2003**	**2004**	**2005**	**2006**	**2007**	**2008**	**2009**	**2010**	**2011**	***2020***	***2030***
**Occupational PA**																			
All Adults	112.8	110.3	*98.9*	87.5	*93.5*	99.4	100.4	100.8	100.4	102.4	101.8	117.2	116.1	118.4	115.6	116.9	114.4	*112.0*	*107.7*
Males	137.4	132.6	*119.5*	106.4	*112.2*	118.0	117.9	116.4	114.2	117.4	118.7	141.3	135.3	138.5	133.1	138.8	135.0	*129.1*	*121.5*
Females	89.8	89.4	*79.3*	69.3	*75.3*	81.4	83.4	85.8	86.8	88.0	85.7	95.4	97.8	99.3	98.9	96.2	95.4	*95.6*	*94.0*
**Domestic PA**																			
All Adults	90.5	89.3	*80.5*	71.7	*76.7*	*81.6*	*81.0*	*80.3*	*79.6*	*78.9*	*78.2*	71.3	77.9	77.3	80.5	*74.8*	*74.1*	*78.4*	*80.6*
Males	45.7	47.2	*42.3*	37.3	*39.8*	*42.3*	*42.0*	*41.7*	*41.4*	*41.1*	*40.8*	37.5	38.6	39.5	44.8	*39.3*	*39.0*	*44.5*	*48.9*
Females	132.3	128.6	*116.7*	104.9	*111.8*	*118.7*	*117.7*	*116.6*	*115.6*	*114.6*	*113.5*	103.0	115.0	113.1	114.4	*108.4*	*107.3*	*111.3*	*112.3*
**Travel PA**																			
All Adults	77.9	72.8	*68.5*	64.3	*67.9*	71.6	57.9	59.5	57.4	54.8	57.1	*51.8*	*49.7*	*47.6*	*45.4*	*43.3*	*41.1*	*24.0*	*2.6*
Males	81.7	75.5	*72.1*	68.8	*71.4*	74.1	59.7	61.5	60.0	57.2	59.3	*53.6*	*51.3*	*49.0*	*46.7*	*44.4*	*42.1*	*23.7*	*0.7*
Females	74.5	70.3	*65.1*	60.0	*64.6*	69.3	56.3	57.7	55.0	52.6	55.0	*50.1*	*48.1*	*46.1*	*44.1*	*42.1*	*40.1*	*24.2*	*4.2*
**Active leisure PA**																			
All Adults	1.7	1.8	*2.2*	2.6	*2.7*	2.8	2.8	2.9	2.9	2.5	2.6	2.1	*2.4*	*2.4*	2.0	2.3	2.1	*2.1*	*2.0*
Males	2.4	2.5	*3.0*	3.5	*3.8*	4.0	3.8	3.8	4.0	3.6	3.7	2.8	*3.2*	*3.2*	2.7	3.0	2.7	*2.5*	*2.1*
Females	1.0	1.1	*1.3*	1.6	*1.7*	1.7	1.8	2.0	1.7	1.5	1.5	1.4	*1.6*	*1.6*	1.4	1.6	1.6	*1.8*	*2.0*
**Total PA**																			
All Adults	282.9	274.1	*250.1*	226.1	*240.8*	255.5	242.1	243.5	240.3	238.7	239.7	242.4	246.0	245.7	243.5	237.2	231.7	*216.5*	*193.0*
Males	267.2	257.8	*236.9*	216.1	*227.2*	238.3	223.4	223.4	219.6	219.3	222.5	235.2	228.4	230.3	227.3	225.5	218.9	*199.7*	*173.1*
Females	297.6	289.3	*262.5*	235.8	*253.4*	271.1	259.2	262.0	259.1	256.8	255.6	249.9	262.5	260.1	258.8	248.4	244.4	*232.7*	*212.4*

^a^Forecasting based on using 2006–2011 slopes are presented for all domains for 2020 and 2030.

^b^Italicized values were determined from linear interpolation for domestic PA from 2000–2005 and 2010–2011, for travel PA from 2006–2011, for active leisure PA for 2007 and 2008, and for all domains in 1997 and 1999.

**Table 3:** **Average hours per week for activity domains from RLMS 1995 to 2011**^**a**^

**Activity domain**	**Average hours per week by survey year**														
	**1995**	**1996**	**1998**	**2000**	**2001**	**2002**	**2003**	**2004**	**2005**	**2006**	**2007**	**2008**	**2009**	**2010**	**2011**
**Occupational PA**															
All Adults	31.1	30.5	24.5	27.6	28.1	28.3	28.0	28.5	28.3	33.3	32.9	33.2	32.6	33.2	32.7
Males	34.7	33.4	27.1	29.8	30.1	29.7	29.0	29.6	29.9	36.2	34.6	35.0	33.8	35.6	34.8
Females	27.8	27.9	21.9	25.5	26.2	26.9	27.1	27.5	26.7	30.8	31.2	31.6	31.5	31.0	30.7
**Domestic PA**															
All Adults	29.0	28.6	23.6	*26.5*	*26.3*	*26.2*	*26.0*	*25.8*	*25.6*	23.3	25.8	25.7	26.2	*24.8*	*24.6*
Males	13.5	13.9	11.5	*12.8*	*12.8*	*12.8*	*12.8*	*12.7*	*12.7*	11.8	12.3	12.7	13.8	*12.6*	*12.5*
Females	43.6	42.3	35.2	*39.4*	*39.1*	*38.8*	*38.5*	*38.2*	*37.9*	34.1	38.5	38.1	38.1	*36.3*	*36.0*
**Travel PA**															
All Adults	26.0	24.3	21.4	23.9	19.3	19.8	19.1	18.3	19.0	*17.3*	*16.6*	*15.9*	*15.1*	*14.4*	*13.7*
Males	27.2	25.2	22.9	24.7	19.9	20.5	20.0	19.1	19.8	*17.9*	*17.1*	*16.3*	*15.6*	*14.8*	*14.0*
Females	24.8	23.4	20.0	23.1	18.8	19.2	18.3	17.5	18.3	*16.7*	*16.0*	*15.4*	*14.7*	*14.0*	*13.4*
**Active leisure PA**															
All Adults	0.3	0.3	0.4	0.5	0.5	0.5	0.5	0.4	0.4	0.3	*0.4*	*0.4*	0.3	0.4	0.3
Males	0.4	0.4	0.6	0.6	0.6	0.6	0.7	0.6	0.6	0.4	*0.5*	*0.5*	0.4	0.5	0.5
Females	0.1	0.2	0.2	0.3	0.3	0.3	0.3	0.2	0.2	0.2	*0.3*	*0.3*	0.2	0.3	0.2

^a^Italicized values were determined from linear interpolation for domestic PA from 2000–2005 and 2010–2011, for travel PA from 2006–2011, for active leisure PA for 2007 and 2008.

Over a relatively short period of time (1995 to 1998), notable declines in occupational, domestic and travel PA were found (Table 2; Figure 1a-1c). From 1995 to 1998, occupational PA dropped by 22% (112.8 to 87.5 MET-hours per week), domestic PA fell by 21% (90.5 to 71.7 MET-hours per week), and travel PA dropped by 17% (77.9 to 64.3 MET-hours per week). Total PA MET-hours per week declined by 51.1 MET-hours per week among males and by 61.8 MET-hours per week among females from 1995 to 1998. In the ensuing years (1999–2005), PA increased and then stabilized across all domains. From 2006 and beyond, increases in occupational PA and declines in travel PA were seen.

Among the subset of adults in 2001 for whom there was data on sedentary leisure (television and video watching), the average hours spent per week was 18.5 and average MET-hours per week was 24.1. MET-hours per week of sedentary leisure was higher among male versus female adults in 2001 (24.9 and 23.4, respectively). Among the subset of adults in 2002, the average hours spent per week in sedentary activity was 20.7 and the average MET-hours per week was 26.9. Again, MET-hours per week of sedentary leisure was higher among male versus female adults (28.0 and 26.0, respectively). However, because the measure of sedentary leisure was limited to television and video watching, these are likely underestimates. In addition, with only two Rounds of data available for this measure for a subset, we were unable to reliably interpolate for the years prior and after.

Annualized changes, total % changes, and annualized % changes between time 1 and time 2 for all PA domains using observed data are shown in Table 4. The greatest changes were in travel PA (i.e., largest annual and relative declines in travel PA); these declines were consistent among males and females. Females also experienced declines in domestic PA over time, with a 13.5% relative decline in MET-hours per week from 1995 to 2009. Annualized changes for occupational and active leisure PA were less distinct among all adults and by gender.

**Table 4:** **Observed changes in occupational, domestic, travel and active leisure PA (MET-hrs/week) for adults (18-60 y)**^**a**^

**PA domain (Survey years)**	**MET-hours per week at time 1**	**MET-hours per week at time 2**	**Annualized change between time 1 and time 2**	**Total % change between time 1 and time 2**	**Annualized % change between time 1 and time 2**
Occupational PA (1995–2011)					
All Adults	112.8	114.4	0.1	1.4	0.1
Males	137.4	135.0	−0.1	−1.7	−0.1
Females	89.8	95.4	0.3	6.2	0.4
Domestic PA (1995–2009)					
All Adults	90.5	80.5	−0.7	−11.0	−0.8
Males	45.7	44.8	−0.1	−2.0	−0.1
Females	132.3	114.4	−1.3	−13.5	−1.0
Travel PA (1995–2005)					
All Adults	77.9	57.1	−2.1	−26.8	−2.7
Males	81.7	59.3	−2.2	−27.5	−2.7
Females	74.5	55.0	−1.9	−26.2	−2.6
Active leisure PA (1995–2011)					
All Adults	1.7	2.1	<0.1	27.6	1.7
Males	2.4	2.7	<0.1	13.0	0.8
Females	1.0	1.6	<0.1	63.2	4.0

^a^Note: Total PA is not presented due to different baseline years by PA domain.

Forecasted changes in occupational, domestic, travel and active leisure PA (MET-hrs/week) for Adults (18-60 y) for 2020 and 2030 are shown in Table 5. We found that depending on the approach used, the forecasted PA levels for 2020 and 2030 can vary substantially. Forecasted total PA and travel PA values were very similar between using the 2006–2011 slope and using the 2008–2011 slope. Because the 2006–2011 slope provided the middle value for occupational PA, which was the main contributor to total PA, we chose to focus on this value. However, we do note the difference.

**Table 5:** Forecasted changes in occupational, domestic, travel and active leisure PA (MET-hrs/week) for adults (18-60 y)

**PA domain**	**MET-hrs/wk in 1995**	**MET-hrs/wk in 2020 using 2006–2011 slope**	**MET-hrs/wk in 2020 using 2008–2011 slope**	**MET-hrs/wk in 2020 using 3-year moving averages**	**Total % change from 1995 to 2020**^**a**^	**MET-hrs/wk in 2030 using 2006–2011 slope**	**MET-hrs/wk in 2030 using 2008–2011 slope**	**MET-hrs/wk in 2030 using 3-year moving averages**	**Total % change from 1995 to 2030**^**a**^
**Occupational PA**									
All Adults	112.8	112.0	106.0	115.5	0.8	107.7	95.1	115.5	−1.1
Males	137.4	129.1	131.7	135.8	−3.6	121.5	126.8	135.8	−6.3
Females	89.8	95.6	83.7	96.3	6.8	94	69.2	96.3	5.9
**Domestic PA**									
All Adults	90.5	78.4	62.1	75.4	−15.0	80.6	46.9	75.4	−13.8
Males	45.7	44.5	34.1	40.1	−7.4	48.9	27.1	40.1	−2.6
Females	132.3	111.3	88.7	108.9	−16.8	112.3	65.5	108.9	−16.4
**Travel PA**									
All Adults	77.9	24.0	24.0	42.6	−57.3	2.6	2.7	42.6	−71.0
Males	81.7	23.7	23.7	43.6	−58.8	0.7	0.7	43.6	−72.9
Females	74.5	24.2	24.2	41.4	−56.0	4.2	4.2	41.4	−69.4
**Active leisure PA**									
All Adults	1.7	2.1	1.7	2.2	28.8	2.0	1.2	2.2	25.8
Males	2.4	2.5	1.8	2.8	9.0	2.1	0.6	2.8	0.8
Females	1.0	1.8	1.7	1.6	77.4	2.0	1.8	1.6	87.9
Total PA									
All Adults	282.9	216.5	216.5	235.7	−20.1	192.9	193.0	235.7	−24.2
Males	267.2	199.8	199.7	222.3	−21.0	173.2	173.1	222.3	−26.0
Females	297.6	232.9	232.7	248.2	−19.2	212.5	212.4	248.2	−22.6

^a^The 2020 or 2030 value used in the total % change measure was the midpoint value from the 2006–2011 slope and moving averages calculations.

Occupational PA fell between 1995 and 2020/2030 when using the 2006–2011 (and 2008–2011) slope to forecast, but rose slightly when using the three-year moving averages. For domestic PA, there was a decline regardless of the approach used, but the decline using the 2008–2011 slope was the greatest, while the decline using the 2006–2011 slope was the gentlest. In addition, while the forecasted travel PA using all approaches showed a decline since 1995, the three-year moving averages approach yielded the smallest decline. The total % change presented therefore provides the mid-point estimate of the relative forecasted change between 1995 and 2020 or 2030. In looking at these, we see that the greatest declines are forecasted to occur in travel PA; these declines are consistent among males and females. Declines in domestic PA are also expected, with declining rates being greater among females versus males. Occupational PA is forecasted to decrease among males, but increase among females. Meanwhile, little change is expected in MET-hours per week from active leisure PA for males, but females are forecasted to increase their active leisure PA although the absolute level is still very low.

## Discussion

Using nationally representative data from a country experiencing major economic transitions, we provide a comprehensive look at PA patterns and projections in Russian adults over a 16-year time period. Early in this time period (1995–1998), we document how a significant financial crisis coincided with domain-specific reductions in PA. In the ensuing years of economic recovery, our findings show corresponding increases in PA across all domains. Overall declines in total PA (namely in domestic and travel PA) are consistent with international trends characterized by more modern lifestyles and economic growth [1,9]. Projections in PA for 2020 and 2030 indicate troubling trends if no action is taken, thus domain-specific initiatives to prevent further PA declines are imperative.

PA reductions from 1995 to 1998 occurred when Russia was experiencing decreased economic productivity, political and economic instability, rising poverty, and other challenges that culminated in the Russian financial crisis of 1998 [17,26,30-32]. Occupational, domestic, and travel PA reached their lowest points in 1998, but later increased and evened out during the period of economic recovery from 1999 to 2006. Effects of the milder recession in 2008–2009 were less notable on PA patterns. Therefore, our findings suggest that trends in domain-specific PA correlate with patterns of economic instability and recovery.

Among male adults, the largest contributor to total PA was occupational PA followed by travel PA. In contrast, domestic PA followed by occupational PA contributed most to total PA among females adults. Total PA was 282.9 MET-hours per week in 1995 and declined to 231.7 in 2011. The greatest relative declines are occurring in travel PA, and female adults are also exhibiting significant declines in domestic PA. The declines in domestic PA among females are concurrent with increases in occupational PA; these trends are likely resultant from more women entering the workforce, women working longer hours, and a shifting of time demands away from the home and toward work. Changes in active leisure PA are less distinct. In comparing these results to past results for the United States, United Kingdom, Brazil, India and China [9], we found that the trends in Russia (excluding occupational PA) are following what has been observed in these five countries. Declines in travel and domestic PA have been well-documented across countries, mainly driven by increases in passive travel and greater access to modern technology for home production activities [23,33-38]. While it is surprising that Russian occupational PA has not declined more, this may reflect to some extent the lack of modernization of the dominant manufacturing sector and the lack of a shift in occupational structure toward a much greater proportion in the service sector found in most higher income countries as income improves significantly [39,40].

Total PA is projected to decrease to 216.5 MET-hours per week in 2020 and to 193.0 MET-hours per week in 2030. The 23.5 MET-hours per week reduction from 2020 to 2030 is roughly equivalent to 3.9 to 7.8 hours of moderate PA. These projections are largely influenced by decreased travel and domestic PA, whereas forecasted occupational and active leisure PA patterns are more stable over time. The more stable occupational PA patterns may be a consequence of this activity reaching a lowest possible limit (bottoming out effect). Stable active leisure PA patterns are expected without time use changes across the domains (e.g., increased active leisure PA requires time use reductions in sedentary activities or in other domains) or with increases in the intensity of active leisure activities. Projected reductions in other domains are highly probable without action.

Development of domain-specific initiatives, particularly for travel and active leisure PA, are needed to promote more active travel and leisure activities. Focusing initiatives in active travel and leisure domains have proven effective in improving PA [33,41-45] and could help counteract projected declines in total PA. Efforts can range from congestion charging schemes to reduce car use, with a resultant increase in cycling and walking for transport and other positive outcomes, such as improved air quality, lower carbon footprint, lower noise pollution and lower congestion [46], to a growing array of transportation options. However, without disincentives to car ownership and use, better active transport infrastructure, and improved mass transit, these changes are not likely to occur.

We faced some data limitations that warrant explanation. First, there was a lack of completeness in the survey questions asked over the various rounds of the RLMS. Specifically, some questions were included in some but not all of the RLMS rounds. Consequently, we had to conduct linear interpolation for domestic PA from 2000–2005 and 2010–2011, for travel PA from 2006–2011, and for active leisure PA for 2007 and 2008. These steps might have affected the precision of our forecasts in particular. Additionally, the RLMS questions on travel PA and sedentary time were limited in terms of the travel modes included and type of sedentary activities. Lastly, the way in which the RLMS collects information about time spent in various domains does not allow for simultaneous activities (e.g., caring for a child while preparing food), and so may overestimate PA. However, for the purposes of understanding trends, so long as the cause and degree of mis-estimation is random and consistent over time, we do not believe this is a problem.

## Conclusion

Our study provides an initial look at nationally-representative, domain-specific PA patterns and projections for Russian adults over an extended time period marked by major economic change. These results add to earlier work that documents the dramatic global trends in declines in PA and rises in inactivity in the US, UK, Brazil and India [9]. As a populous and aging country, the long term health implications of these trends can be significant. More needs to be done to encourage movement in Russia via investments into infrastructure, interventions and initiatives that promote PA across all domains of living, particularly active travel, active leisure (exercise) as well as certain domestic activities (e.g., gardening). In order for these interventions and initiatives to be effective, they must recognize competing time demands and incorporate strategies promoting increased time and/or intensity spent in active travel, active leisure, and domestic domains.

From a methodological standpoint, the inclusion of time and intensity measures for occupational activities in the RLMS enabled assessment of three distinct approaches for the estimation of MET values for occupational PA. Comparison of Approaches A and B yielded consistent findings, thus supporting the robustness of the widely used approach of assigning MET values to occupations based on the Compendium of Physical Activities. Additional methodological exploration was conducted with respect to PA projections, given the application of three approaches for estimating domain-specific levels of PA in 2020 and 2030. Further study is planned to examine determinants of the PA trends and also to utilize RLMS longitudinal data to compare age, period, and cohort effects of environmental and individual factors on PA behaviors.

## Abbreviations

MET: Metabolic equivalent of tasks; PA: Physical activity; IPAQ: International physical activity questionnaire; GPAQ: Global physical activity questionnaire; RLMS: Russia Longitudinal Monitoring Survey.

## Competing interests

The work presented in this paper was commissioned by funds from Nike, Inc. The authors completed the manuscript independently with assistance of reviewers.

## Authors’ contributions

BMP designed the physical activity measures for the RLMS. TDW conducted the data cleaning, analyses and drafted the manuscript. SWN led the study methods, approach and data interpretation, drafted and revised the manuscript. BMP is the co-PI of the RLMS study and participated in the methods and approach, reviewed and revised the manuscript. All authors read and approved the final manuscript. None of the authors has conflicts of interest with respect to this manuscript.
